# Clinical and Molecular Effects of GnRH Agonist and Antagonist on The Cumulus Cells in The *In Vitro* Fertilization Cycle

**DOI:** 10.22074/IJFS.2020.136161.1012

**Published:** 2021-06-22

**Authors:** Saeid Azizollahi, Maryam Bagheri, Fedyeh Haghollahi, Seyyede Momeneh Mohammadi, Batool Hossein Rashidi

**Affiliations:** 1 Vali-e-Asr Reproductive Health Research Center, Tehran University of Medical Sciences, Tehran, Iran; 2 Cell Therapy and Regenerative Medicine Comprehensive Center, Kerman University of Medical Sciences, Kerman, Iran; 3 Department of Anatomical Sciences, School of Medicine, Zanjan University of Medical Sciences, Zanjan, Iran

**Keywords:** Apoptosis, Cumulus Cells, GnRH Antagonist

## Abstract

**Background:**

Gonadotropin-releasing hormone (GnRH) analogues have been extensively utilized in the ovarian stimulation cycle for suppression of endogenous rapid enhancement of luteinizing hormone (LH surge). Exclusive properties and functional mechanisms of GnRH analogues in in vitro fertilization (IVF) cycles are clearly described. This study was performed to evaluate clinical and molecular impacts of the GnRH agonist and antagonist protocols in IVF cycles. For This purpose, gene expression of cumulus cells (CCs) as well as clinical and embryological parameters were evaluated and compared between two groups (GnRH agonistand antagonist) during the IVF cycle.

**Materials and Methods:**

Twenty-one infertile individuals were enrolled in this study. Subjects were selected from two groups of GnRH agonist(n=10) treated patients and GnRH antagonist (n=11) treated individuals. The defined clinical embryological parameters were compared between the two groups. Expression of *BAX, BCL-2, SURVIVIN, ALCAM,* and *VCAN* genes were assessed in the CCs of the participants using the real-time polymerase chain reaction (PCR) technique.

**Results:**

The mean number of cumulus oocyte complex (COC), percentage of metaphase II (MII) oocytes, grade A embryo and clinical parameters did not show noticeable differences between the two groups. *BAX* gene expression in the CCs of the group treated with GnRH agonist was remarkably higher than those received GnRH antagonist treatment (P<0.001). The mRNA expression of *BCL-2* and *ALCM* genes were considerably greater in the CCs of patients who underwent antagonist protocol in comparison to the group that received agonist protocol (P<0.001).

**Conclusion:**

Despite no considerable difference in the oocyte quality, embryo development, and clinical outcomes between the group treated with GnRH agonist and the one treated with antagonist protocol, the GnRH antagonist protocol was slightly more favorable. However, further clinical studies using molecular assessments are required to elucidate this controversial subject.

## Introduction

The gonadotropin-releasing hormone (GnRH) agonist and antagonist protocols are extensively utilized in the ovarian stimulation cycle to inhibit the endogenous rapid increase in the luteinizing hormone (LH surge) levels. The unique properties and functional mechanisms of GnRH analogues in the *in vitro* fertilization (IVF) cycles are well defined (1).

GnRH agonist have a longer half-life and higher potential than native GnRH. They initially stimulate pituitary gonadotrophs and production of follicle-stimulating hormone (FSH) and LH hormones, thereby cause an expected response of gonads (2). In contrast, GnRH antagonist immediately suppress pituitary gonadotropin in the competition with the GnRH receptor, thereby prevent early excitatory phase of agonists. Recently, there have been an increasing interest in using GnRH antagonist in control ovarian hyperstimulation (COH). GnRH antagonist have beneficial effects compared to the GnRH agonist. Most notably they cause fewer follicles and lower daily usage of estradiol, and thus lower incidence of ovarian hyperstimulation syndrome (OHSS), a serious complication which eventually helps the reproductive treatment (3). However, it has been reported that GnRH antagonist administration is along with a reduced live birth rate and an increase in the risk of pregnancy loss, which might be the result of impaired implantation and lower estradiol concentrations on the first day of COH (2). In addition to the pituitary, the role of GnRH in other tissues including ovary, uterus, and placenta have been demonstrated in previous studies. Although the mode of action of GnRH and its analogues are well determined on the pituitary level, its role in the extra pituitary tissues is still not fully understood (4).

GnRH receptors are present on the ovarian epithelial cells, granulosa cells (GCs), and cumulus cells (CCs). CCs are involved in the follicular development, maturity, and quality of the oocyte (5). There is a bidirectional paracrine communication between the CCs and oocytes during folliculogenesis (6). By secreting paracrine markers including growth differentiation factor 9 (GDF-9) and bone morphogenetic protein-15 (BMP-15), the oocyte induces CC gene expression to ensure its development and maturation (7). For this reason, optimal development and the quality of the oocyte can be evaluated by the CC gene expression as a non-invasive method (6). *Versican (VCAN)* and activated leukocyte cell adhesion molecule (*ALCAM*) are expressed in the CCs and contribute to the extracellular matrix (ECM) formation (8). *VCAN*, which is a proteoglycan, is expressed in the GCs after ovulation induction. *VCAN* is cleaved following LH surge by a precise molecular pathway and the cleaved *VCAN*, as the functional form, is observed in the COCs (9). Since important growth factor receptors are attached to this functional form, a change in the *VCAN* expression might also alter COC matrix properties during the oocyte maturation, ovulation, and fertilization (8). *ALCAM* is known as an ECM-related protein. Cell to cell and cell to matrix adhesion may be promoted by *ALCAM* in the reproductive tissues. *ALCAM* has been shown to be expressed in the epithelium and blastocysts and is involved in the implantation process (10). A significant association is reported between the expression of these genes and oocyte quality (11). 

Moreover, it seems that apoptosis of CCs reduces the success rate in IVF (12) and the higher the incidence of apoptotic CCs, the lower the fertilization rate (13). The vital role of programmed cell death in different physiological events of reproduction is well established. For instance, during folliculogenesis, the number of follicles in a follicular cohort primarily diminishes due to the apoptosis of GCs (14). *SURVIVIN* is a member of inhibitors of apoptosis proteins (IAPs) and has an important caspase inhibitory function (15). Critical functions of survivin in folliculogenesis and follicular development are not limited to apoptosis inhibition, but also this protein participates in the regulation of the mitotic spindle checkpoint (16). Follicular development or atresia are regulated by different hormonal and microenvironmental factors (17). AMH, GnRH, androgens, and apoptotic (*BAX, P53, FOXO3*) and anti-apoptotic (*BCL-2, SURVIVIN*) genes are identified as the follicular atretogenic factors (18). The anti-apoptotic role of *BCL-2* against a variety of cell death-inducing factors has been proved in numerous studies. A correlation has been found between apoptosis acceleration and overexpression of *BAX*, as a pro-apoptotic agent (19).

Therefore, the present study was performed to examine the impact of GnRH agonist and antagonist on IVF cycles from clinical and molecular points of view. For this purpose, the oocyte quality, embryo development, CC gene expression, and pregnancy rate were compared between the two groups of patients who received GnRH agonist or antagonist throughout the IVF cycle. 

## Materials and Methods

### Patients and study design

In this study, 21 eligible infertile women undergoing IVF cycle were chosen. This study was conducted in Vali-e-Asr Reproductive Health Research Center, Tehran University of Medical Sciences (Tehran, Iran) from December 2014 to February 2016. Study was approved by Ethics Committee of Tehran University of Medical Sciences (IR.TUMS.VCR.REC.1396.2309). People who agreed to take part in this study signed a consent form. Participants were divided into two groups of subjects, who received either GnRH agonist or GnRH antagonist. Subjects had an equal chance of being in both groups.

The inclusion criteria for the subjects were age<40 years and body mass index (BMI) <30 kg/m^2^ . Furthermore, the cause of undergoing IVF was tubal factor infertility, and according to WHO criteria, partners had normal sperm parameters. The exclusion criteria were as follows: patients with ovarian dysfunction or other endocrinopathies, infertile couples with severe male factor infertility, poor responders, polycystic ovarian syndrome (PCOS), and endometriosis. 

### Study size

The choices of sample size and study duration were based on the primary outcome obtained from the study of Danhua Pu 2011. A sample size of 11 with 80% power was achieved by a Two-Sided One-Sample t test, which can detect an effect size (i.e. mean difference) of 1.9 between the null hypothesis with no mean difference and the alternative hypothesis mean=-1.9 with an assessed standard deviation of 2.4 and alpha= 0.05. The sample size estimation was conducted using PASS 15 software.

### Stimulation protocols

Ten individuals were picked out from the GnRH agonist treated patients that received triptorelin 0.1 mg/day subcutaneously (Decapeptyl, Ipsen, Italy) in the luteal phase of their preceding cycles based on a standardized long protocol. Following gonadotrophin inhibition, which was confirmed by transvaginal ultrasound, the patients received 150-225 IU recombinant FSH (rFSH) (Gonal-F, Merck Serono Laboratories, Switzerland) on the 3^rd^ day of their periods (20). 

Eleven women in the GnRH antagonist group were treated with 150-225 IU/day rFSH subcutaneously beginning on the second day of their monthly periods followed by a single dose adjustment from day 5 of the cycle. Each patient received 0.25 mg/day of cetrorelix (Cetrotide, Serono) on the sixth day of COH according to a fixed protocol (20).

To assess the ovarian response to the stimulation protocol, prior to the injection of the human chorionic gonadotropin (HCG) hormone, the follicle sizes were measured and clinical tests such as serum estradiol and FSH concentration measurements and transvaginal ultrasounds were performed. Serum FSH and estradiol concentrations were measured using immunoassay kits (CALBIOTECH, USA) with an automated multi-analysis system. 

A single dose of HCG (Gonasi HP 5000, AMSA, Italy) 10,000 IU was injected intramuscularly following the observation of at least three follicles with an optimal diameter of 18mm and serum estradiol ≥0.40 nmol. Oocytes were picked up 34-36 hours after HCG administration.

### Evaluation of parameters

Embryological, clinical, and molecular variables were evaluated to compare the effects of GnRH antagonist and agonist. The pregnancy rate and the number of ovarian follicles were evaluated as clinical parameters. 

**Fig.1:**
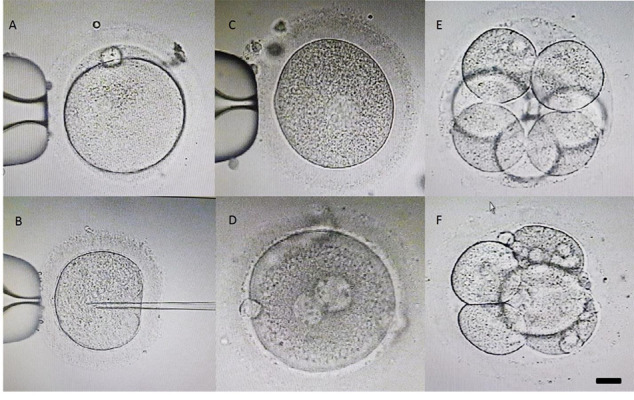
Different stage of oocyte and embryo development that was evaluated in the study. **A.** Metaphase II (MII), **B. **Metaphase I (MI), and **C.** Germinal vesicle (GV) oocyte. **D. **2 pronuclei (2-PN), **E.** 7-cell embryos with less than 10% fragmentation (graded as A), and** F.** 7 cells with more than 10% fragmentation (scale bar: 20 µm)

For evaluation of oocyte competence, the percentage of metaphase II (MII) (Fig .1A), metaphase I (MI) (Fig .1B) and germinal vesicle (GV) oocytes (Fig .1C), were calculated. Furthermore, the percentage of 2 pronuclei (2PN, Fig .1D) from the total number of MII retrieved oocyteswere considered as fertilization rate.

The pregnancy rate was evaluated as the percentage of the subjects with positive βHCG test after receiving either the agonist or the antagonist protocol. The number of years that a woman was infertile was considered as the infertility duration. According to the constructor’s instructions, serum prolactin concentrations were measured using an ELISA kit (Calbiotech, USA). The endometrial thickness and ovarian follicle count (number of follicles more than 18 mm) were measured using gynecological ultrasound.

The percentage of 7-cell embryos with less than 10% fragmentation (graded as A, Fig .1E) and the percentage of embryos with at least 7 cells having >10% fragmentation (graded as AB, Fig .1F) from the total number of fertilized oocytes on day 3 after insemination were assessed and compared between the two groups.

**Table 1:** Primer sequences used in quantitative real time polymerase chai reaction


Gene	Primer sequence (5´-3´)	Annealing temperature (ºC)	Gene

*ALCAM*	F: CTGGCAGTGGAAGCGTCATA	55	189
	R: CGTCTGCCTCATCGTGTTCT		
*VCAN*	F: TCCTCGCAGAAACTGCATCA	59	231
	R: CCCAGGGCTTCTTGGTACTG		
*SURVIVIN*	F: AGGACCACCGCATCTCTACA	55	188
	R: TTTCCTTTGCATGGGGTCGT		
*BAX*	F: GTCTTTTTCCGAGTGGCAGC	55	251
	R: GGAGACAGGGACATCAGTCG		
*BCL-2*	F:GGGAGGATTGTGGCCTTCTT	59	286
	R: ACTTGTGGCCCAGATAGGCA		
*Β-ACTINE*	F: GTCATTCCAAATATGAGATGCGT	60	121
	R: GCTATCACCTCCCCTGTGTG		


For the molecular investigation, expression of *VCAN, ALCAM, SURVIVIN, BAX* and *BCL-2* genes were evaluated in the CCs. 

### Collection and isolation of cumulus cells

The retrieved COCs were washed several times in commercial human tubal fluid (HTF Lonza, Verviers, Belgium) in order to eliminate any blood cells, GCs, and debris contamination. Then, they were incubated in the fertilization medium for 5 minutes (Universal IVF medium, Medicul, Denmark). CCs samples were mechanically dissected less than 1 hour after OPU. Isolation of CCs was performed by washing these cells in the culture medium and centrifugation 10 minutes at 250 × g several times (21). Then, the cells were pooled and preserved by rapid freezing just after dissection and prior to the RNA extraction.

### RNA extraction and real-time polymerase chain reaction

CC RNA extraction was performed using Arcturus Pico Pure RNA Isolation Kit (Applied Biosystems, USA) based on the manufacturer’s instructions (from ~ 4 ng pooled oocyte to 100 ng of CCs, and 3 repetitions for this experiment). DNase I (Fermentas, St. Leon-Rot, Germany) was used three times to eliminate genomic DNA contaminations. The purified RNA was used for cDNA synthesis using oligo dT primers (Applied Biosystems, Foster City, CA) prior to real-time polymerase chain reaction (PCR) (22).

The primers were designed to the human sequence of *VCAN, ALCAM, SURVIVIN, BAX *and *BCL-2 *genes using the Gene Runner (version 3) and Primer Express (version 3.05), and were blasted in http://www.ncbi.nlm. nih.gov/BLASt/. The primer characteristics are presented in Table 1. 

Real-time PCR was accomplished with the SYBR Green Reagent (Applied Biosystems, USA) using ABI PRISM 7300 Analyzer (Applied Biosystems, USA). The PCR cycle was repeated for 45-55 cycles. The Q-PCR reaction was carried out at least three times using specific primers. The quantification of 5 genes was evaluated using the comparison with the housekeeping gene, beta-actin. Finally, 2^−∆∆CT^ technique was used for comparative quantification between the two groups. 

### Statistical analysis

Data were analysed using IBM SPSS Statistics software (version 25, IBM SPSS Statistics, Armonk, USA) and the graphs were drawn by GraphPad (Prism) (version 8, https://www.graphpad.com). Normality of the numeric variables was checked and confirmed by Kolmogorov-Smirnov test and measures of distribution including skewness and kurtosis were within ± 1.5 and ± 2, respectively. Data are presented as the mean (SD) and frequency (percent) for numeric normal and categorical variables, respectively. Comparisons of the variables between the groups were conducted by Independent Samples t test. The assumption of the homogeneity of the variances were assessed by Levene’s test, and Welch correction was used when the assumption was not satisfied. For comparing the categorical variables between the two groups, Pearson Chi-square test with exact P value was utilized. In all analyses, a P<0.05 indicates statistically significant.

## Results

Clinical characteristics of the woman in different groups are shown in Table 2. No significant difference was observed in the infertility duration, age, BMI and hormonal levels between the two groups. In addition, the number of the dominant ovarian follicles, endometrial thickness, and pregnancy rate were not different significantly between the groups.

### Embryological assessments

In GnRH antagonist group, the mean number of obtained COCs was higher than the GnRH agonist group, which was not statistically significant (P=0.14, Table 3). In order to compare the oocyte nuclear maturity, the percentages of MII, MI, and GV oocytes were calculated and compared between the groups. As shown in Table 3, MII percentage is clinically higher in the GnRH antagonist group compared to the agonist group (84.8 ± 20 vs. 78.6 ± 27.6, P=0.57). No considerable difference was found in the percentage of MI and GV oocytes between the two groups (12.6 ± 17.8 vs. 9.2 ± 16.2, and 5.6 ± 9.6 vs 5.5 ± 8.9, P=0.65 and P=0.99, respectively). Moreover, a statistically significant difference was observed in the percentage of 2 PN between the GnRH agonist and GnRH antagonist groups (54.5 ± 19.2 vs. 72.5 ± 9.1, respectively, P<0.05). Finally, the percentage of grade A and AB embryos from the total number of fertilized oocytes were compared in each group. No significant difference was found in the percentage of type A (52.9 ± 34.3 vs. 55.5 ± 29.6%, P=0.24 and AB embryo (21.2 ± 20 vs. 22.9 ± 25%, P=0.87) between the GnRH agonist and GnRH antagonist groups.

### Molecular evaluation

**Fig.2:**
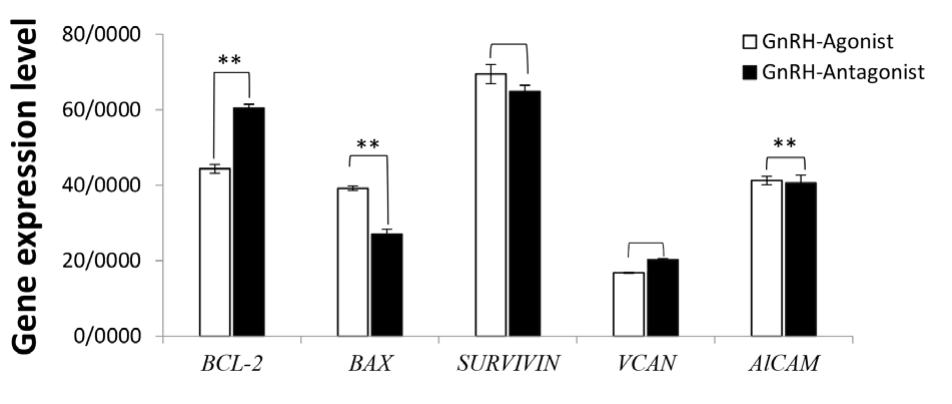
Relative gene expression of apoptotic and developmental gens by real time polymerase chain reaction (PCR). **; Show significant difference.

As shown in Figure 2, the relative *BAX* gene expression in the CCs of patients that received GnRH agonist was significantly higher than those with GnRH antagonist treatment (39.1 ± 2 vs. 27.01 ± 4.2, respectively, P<0.001). Furthermore, expression of *BCL-2* was higher in the CCs of the patients received GnRH antagonist against those who received GnRH agonist (61.4 ± 2.2 vs. 44.3 ± 4.2, P<0.001). The *ALCAM* expression was significantly different between the GnRH agonist and antagonist groups (16.8 ± 0.6 vs. 22.2 ± 1.3, respectively, P<0.001). No significant difference was seen in the expression of *VCAN* (40.5 ± 7.9 vs. 41.8 ± 6.7, P=0.789) and *SURVIVIN* (64.8 ± 6 vs. 66.9 ± 7.6, P=0.131) between the GnRH agonist and antagonist groups, respectively (Fig .2). 

**Table 2:** Clinical characteristics and pregnancy outcomes of woman receiving GnRH agonist or GnRH antagonist


Evaluated parameter	COH type	Mean	SD	SE	Mean difference	95% CI lower	95% CI upper	P value^#^

Age (Y)	Agonis‌t	30.90	4.65	1.47	0.70	-3.43	4.83	------
	Antagonis‌t	30.20	4.13	1.31				
BMI (kg/m^2^)	Agonis‌t	25.63	3.03	1.36	-0.39	-4.26	3.47	-------
	Antagonis‌t	26.03	3.25	1.08				
Infertility duration (Y)	Agonis‌t	6.60	6.23	2.79	-38.70	-162.25	84.85	--------
	Antagonis‌t	45.30	125.42	39.66				
Prolactin (U/L)	Agonis‌t	10.71	7.45	4.30	-47.96	-181.94	86.03	-------
	Antagonis‌t	58.67	102.17	32.31				
FSH (U/L)	Agonis‌t	7.95	1.34	0.95	-64.85	-398.24	268.54	-------
	Antagonis‌t	72.80	199.96	66.65				
Es‌tradiol (ng/L)	Agonis‌t	31.97	21.71	12.53	-76.22	-133.26	-19.19	--------
	Antagonis‌t	108.19	71.51	22.61				
Follicular number	Agonis‌t	12.50	8.66	4.33	1.70	-5.65	9.05	0.624
	Antagonis‌t	10.80	4.29	1.36				
Endometrial thickness (mm)	Agonis‌t	9.75	0.96	0.48	1.89	-0.68	4.46	0.135
	Antagonis‌t	7.86	2.23	0.71				
					Agonis‌t	Antagonis‌t	Pearson Chi-Square (1)	Exact P value
							
Pregnancy	Positive	Count			0	3	3.938	0.114
		% within COH type			0.0%	75.0%		
	Negative	Count			3	1		
		% within COH type			100.0%	25.0%		


GnRH; Gonadotropin-releasing hormone, BMI; Body mass index, FSH; Follicle-stimulating hormone, COH; Control ovarian hyperstimulation, CI; Confidence interval, #; P value from the independent samples t test. In all variables, equal variances assumed based on the results from the Levene's Test for Equality of Variances (All P>0.05).

**Table 3:** Comparison of embryological parameters between GnRH agonist and GnRH antagonist groups


Evaluated parameter	COH type	Mean	SD	SE	Mean difference	95% CI lower	95% CI upper	P value^#^

COC number	Agonis‌t	9.3	5.9	1.87	-5.30	-12.55	1.95	0.142
	Antagonis‌t	14.6	9.1	2.90				
GV (%)	Agonis‌t	5.6	9.6	3.07	0.01	-8.77	8.79	0.998
	Antagonis‌t	5.5	8.9	2.84				
MI (%)	Agonis‌t	12.6	17.8	5.64	3.46	-12.57	19.48	0.656
	Antagonis‌t	9.2	16.2	5.14				
MII (%)	Agonis‌t	78.6	27.6	8.79	-6.18	-28.98	16.62	0.576
	Antagonis‌t	84.8	20.1	6.37				
PN (%)	Agonis‌t	54.5	19.2	6.08	-18.08	-32.23	-3.93	0.015^*^
	Antagonis‌t	72.5	9.1	2.90				
8-Cell (%)	Agonis‌t	52.9	34.3	10.87	17.35	-12.80	47.50	0.242
A-quality	Antagonis‌t	55.5	29.6	9.37				
8-Cell (%)	Agonis‌t	21.2	20.8	6.59	-1.67	-23.37	20.03	0.873
AB-quality	Antagonis‌t	22.9	25.1	7.96				


COC; Cumulus oophorus complex, GV; Germinal vesicle, MI; Metaphase I, MII; Metaphase II, PN; Pro nucleus, CI: Confidence interval, ^#^ ; P value from the independent samples t test, and ^*^ ; Significant P<0.05. The Levene's Test for Equality of Variances showed that the assumption was not satisfied for MI, MII, PN (all P<0.05).

## Discussion

This study showed that *BAX* gene expression in the CCs of patients treated with GnRH agonist was higher than those treated with GnRH antagonist. Furthermore, mRNA expression of *BCL-2* and *ALCM* genes were considerably greater in the CCs of the antagonist group in comparison to the agonist group. The gene expression of CCs in the individuals treated with assisted reproductive technology (ART) have been evaluated in numerous previous studies (23). To best of our knowledge, this is the first study to investigate the effect of GnRH analogues on CC gene expression.

The correlation between apoptosis of CCs and ART outcome has been demonstrated in numerous studies (23). Clavero et al. (24) reported that the apoptosis rate of the GCs is not associated with the oocyte maturity, quality, and pregnancy outcomes. However, Lee et al. (25) found a strong correlation between the apoptosis of CCs and poor oocyte quality. Moreover, up-regulation of pro-apoptotic genes and downregulation of anti-apoptotic genes in the CCs of the non-early cleavage embryos have been previously described (26). It was shown that survivin plays an essential role in the function of GCs and the inhibition of apoptosis (15). In addition, a positive relationship has been observed between the *SURVIVIN* gene expression in the GCs and pregnancy rate (27). According to a study by Assou et al. (28), the overexpression of *BCL-2* is associated with pregnancy outcomes. The present study indicated that the relative expression of *BCL-2* is higher in the GnRH antagonist group as compared to the agonist group. Moreover, *BAX* was overexpressed in the GnRH agonist group as compared to the antagonist group. Furthermore, we found no positive correlation between the expression of apoptotic genes and oocyte quality, embryo development, and pregnancy outcome. 

The effect of different protocols of GnRH analogues on the ART cycle is controversial (29). Similar results have been reported by Kara et al. (30) regarding the serum progesterone and estradiol levels and the pregnancy rate. Prapas et al. (31) reported positive effects of GnRH antagonist on the live birth rate as well as embryologic and clinical outcomes. Furthermore, higher quality of blastocysts have been noticed in the recurrent implantation failure (RIF) patients that received GnRH antagonist compared to those receiving agonist treatment (32). Contrary to the mentioned study, de Souza Jordão et al. (33) revealed a higher total oocyte number and quality, more embryo development, higher implantation rate, and better pregnancy outcomes following GnRH agonist administration. A recent meta-analysis showed an equal pregnancy rate, endometrial thickness, live birth rate, and cancelation rate after the use of GnRH agonist and antagonist in normal-responder patients (34). Although the clinical and embryological results of our study are not consistent with the aforementioned articles, the molecular findings are compatible. This contradiction may be due to different and incomparable sample sizes.

Two of the five genes that were found to be expressed during oocyte maturation were analyzed in the present study (*ALCAM, VCAN*) (8). A negative correlation was explained between *VCAN* expression level and the percentage of mature oocyte formation. Moreover, decreased *VCAN* expression was shown in the CCs of the subjects with mature oocytes (35). In our study, a relatively lower expression of *VCAN*, which was not statistically significant, was observed in the GnRH antagonist group. *ALCAM* is known as an ECM-related protein. Cell to cell and cell to matrix adhesion may be promoted by *ALCAM* in the reproductive tissues. *ALCAM* has been shown to be expressed in the epithelium and blastocysts and it has an important role in the implantation process (36). A previous study stated expression of *ALCAM* in the CCs and GCs during the ovulatory response (37). Moreover, a positive correlation between the *ALCAM* expression and proper embryo cleavage has been indicated. It was also introduced as a promising new marker for non-invasive embryo selection (35). We also found a significantly higher *ALCAM* expression in the CCs of the GnRH antagonist-treated group in comparison to the agonist group. 

## Conclusion

Despite no considerable difference in the oocyte quality, embryo development, and clinical outcomes between GnRH agonist and antagonist, the GnRH antagonist protocol is more favorable considering our molecular findings. In fact, further molecular studies should be performed on this controversial subject to define the exact effect of GnRH analogues on the reproductive system and to identify any advantage or superiority between the GnRH agonist and antagonist protocols. 
